# Proliferation and aneusomy predict survival of young patients with astrocytoma grade II

**DOI:** 10.1038/sj.bjc.6601067

**Published:** 2003-07-01

**Authors:** P H Wessels, A H N Hopman, B Kubat, A G H Kessels, E W Hoving, M I J Ummelen, F C S Ramaekers, A Twijnstra

**Affiliations:** 1 Department of Neurology, Research Institute Growth and Development (GROW), University Hospital Maastricht, The Netherlands; 2 Department of Molecular Cell Biology, Research Institute Growth and Development (GROW), University Maastricht, The Netherlands; 3 Department of Pathology, Research Institute Growth and Development (GROW), University Hospital Maastricht, The Netherlands; 4 Department of Clinical Epidemiology, Research Institute Growth and Development (GROW), University Hospital Maastricht, The Netherlands; 5 Department of Neurosurgery, University Hospital Groningen, The Netherlands

**Keywords:** astrocytoma grade II, prognosis, proliferation, aneusomy, Ki-67 index, *in situ* hybridisation

## Abstract

The clinical course of astrocytoma grade II (AII) is highly variable and not reflected by histological characteristics. As one of the best prognostic factors, higher age identifies rapid progressive A II. For patients over 35 years of age, an aggressive treatment is normally propagated. For patients under 35 years, there is no clear guidance for treatment choices, and therefore also the necessity of histopathological diagnosis is often questioned. We studied the additional prognostic value of the proliferation index and the detection of genetic aberrations for patients with A II. The tumour samples were obtained by stereotactic biopsy or tumour resection and divided into two age groups, that is 18–34 years (*n*=19) and ⩾35 years (*n*=28). Factors tested included the proliferation (Ki-67) index, and numerical aberrations for chromosomes 1, 7, and 10, as detected by *in situ* hybridisation (ISH). The results show that age is a prognostic indicator when studied in the total patient group, with patients above 35 years showing a relatively poor prognosis. Increased proliferation index in the presence of aneusomy appears to identify a subgroup of patients with poor prognosis more accurately than predicted by proliferation index alone. We conclude that histologically classified cases of A II comprise a heterogeneous group of tumours with different biological and genetic constitution, which exhibit a highly variable clinical course. Immunostaining for Ki-67 in combination with the detection of aneusomy by ISH allows the identification of a subgroup of patients with rapidly progressive A II. This is an extra argument not to defer stereotactic biopsy in young patients with radiological suspicion of A II.

Controversy exists with respect to optimal treatment protocols for low-grade diffuse astrocytoma (astrocytoma WHO grade II; A II) because prospective studies comparing treatment strategies are rare (Karim *et al*, 1996,^2002^; Shaw *et al*, 2002). Another reason for this controversy is that the interval after which malignant progression of A II occurs is difficult to predict. Clinical factors that may correlate with survival include those related to patient age, presenting symptoms, duration of symptoms, performance status, tumour volume, extent of resection, and timing and dose of radiotherapy (Berger *et al*, 1994; Lote *et al*, 1997). Patient age is the single consistent prognostic factor in these retrospective studies. Patient age under 35 years (Shaw *et al*, 1989; Arienti *et al*, 2001), or under 40 years in other series (Bauman *et al*, 1999) is often associated with a prolonged survival. Since the benefits of early treatment have to be balanced against the possibility of long-term side effects from radiation therapy, patient age is decisive in current treatment protocols for A II. For patients under 35 years of age, the benefit of early and aggressive treatment has never been proven sufficiently and therefore treatment is often deferred (Vecht, 1993). As a result, in young patients also a controversy exists concerning the usefulness of an immediate histological diagnosis, involving stereotactic biopsy (Recht *et al*, 1992).

Ultimately the majority of A II progresses to high-grade astrocytomas (astrocytoma WHO grade III and grade IV; A III/A IV), which is characterised by an increase in proliferation activity and an accumulation of genetic abnormalities. Proliferation and cytogenetic markers may therefore identify rapid progressive A II. Increased proliferation activity correlates with shorter survival in most series of astrocytomas, although the number of A II was often too small for separate analyses (Sallinen *et al*, 1994; Korkolopoulou *et al*, 1997). Numerical chromosomal aberrations have been reported in astrocytomas, such as aneusomy 1, trisomy 7 and monosomy 10. It has been shown that trisomy 7 correlates with shorter survival of A II patients (Wessels *et al*, 2002).

In the underlying study, the prognostic value of the proliferation (Ki-67) index and the detection of numerical aberrations for chromosomes 1, 7, and 10 was evaluated. The correlation of ;these parameters with survival analysis was performed for patients aged 18–34 years and ⩾35 years to assess whether these parameters allow the identification of rapid progressive A II in young patients.

## MATERIALS AND METHODS

### Patient material

Tissue specimens from 47 adult patients diagnosed with supratentorial astrocytoma grade II were collected from the data files of the Departments of Pathology of the University Hospitals of Maastricht and Groningen, and the Atrium Hospital in Heerlen, The Netherlands. Histopathological revision according to the WHO classification (Kleihues and Cavenee, 2000) revealed 44 fibrillary astrocytomas and three gemistocytic astrocytomas (all WHO grade II). Astrocytomas with mitotic figures were not included in this series. In order to diminish the probability of sampling error A II in which the neuroradiologists suspected high-grade astrocytoma and which showed radiological characteristics for tumour bleeding as well as extensive contrast enhancement, were not included in this series. Patient records were examined with regard to the first neurological symptoms, radiological findings, neurosurgical procedure, dose and timing of radiotherapy, and survival interval.

Mean and median ages were respectively, 38 and 41 years (range, 18–69 years). The study included 22 women (47%) and 25 men (53%). Seizures were the most frequent presenting symptom (77%), followed by focal neurological deficit (26%), mental changes (15%) and signs of raised intracranial pressure (17%). The median duration of preoperative symptoms was 3 months (range: 1 week–157 months). Most tumours were located in the frontal (51%) and temporal/parietal (45%) lobes and less frequently in the occipital lobe (4%). The majority of patients (*n*=41) underwent neurosurgery immediately after coming for medical attention and had their first neuroimaging test. In six patients, neurosurgical intervention was extended for 36–150 months. No association was found, using Pearson's correlation coefficient, between preoperative interval and proliferation index or chromosomal status. For this reason all cases were included in this study.

Neurosurgical procedures consisted of a biopsy in 29 (62%) and resection in 18 (38%) patients. Radiotherapy was given immediately postoperative in 28 (60%) patients, delayed in 10 (21%) patients, and nine (19%) patients were still not irradiated at the last follow-up.

### Proliferation index: Ki-67 immunohistochemistry

Paraffin sections (5-*μ*m thick) were preincubated in methanol with 0.3% H_2_O_2_. Tissues known to be negative and positive for Ki-67 were used as controls. Antigen retrieval was achieved by incubation with 10 mM citrate buffer (pH 6.0) in a domestic microwave oven at 700 W for 10 min. The sections were incubated with the mouse monoclonal antibody MIB-1 directed against Ki-67 (Immunotech S.A., Marseille, France) at a 1 : 12 solution in PBS containing 4% normal goat serum for 60 min. Subsequently, biotin-labelled horse anti-mouse antibody at a 1 : 200 dilution and avidin–biotin peroxidase complex (Vector Laboratories, Burlingame, CA, USA) were applied for 60 and 45 min, respectively. Peroxidase activity was detected using diaminobenzidine in PBS/ imidazol buffer with 0.02% H_2_O_2_. Nuclei were scored for positivity in 500 cells in each sample in the areas with highest immunopositivity. The mean proliferation (Ki-67) index was 2.7% (range 0.0–9.2%).

### Detection of numerical chromosomal aberrations by *in situ* hybridisation (ISH)

*In situ* hybridisation was performed as described earlier (Hopman and Ramaekers, 1998). Paraffin sections (5-*μ*m thick) were deparaffinised and pretreated in 85% formic acid/0.3% H_2_O_2_ for 20 min. Thereafter the slides were incubated in 1 M sodium-thiocyanate at 80°C for 10 min. Subsequently, proteolytic digestion was performed by 4 mg ml^−1^ pepsin (Sigma Chemical Co, St Louis, MO, USA) for 10 min at 37°C in 0.02 M HCl. Then the slides were fixed in 1% formaldehyde in phosphate-buffered saline (PBS) for 15 min, and rinsed in PBS and double distilled water.

The biotin-labelled DNA probes used in this study were specific for the centromeric regions of chromosome 1 (1q12, pUC 1.77) and chromosome 10 (D10Z1), and the alphoid region of chromosome 7 (p7t1). The probes were hybridised to the target-DNA in a mixture containing 2 × standard saline citrate (SSC), 60% formamide, 10% dextran sulphate, and 0.2 mg ml^−1^ herring sperm DNA. After denaturation at 80°C for 5 min, the slides were incubated overnight at 37°C. Subsequently, the slides were washed twice in 2 × SSC/0.05% Tween at 45°C, and in four × SSC/0.05% Tween at room temperature. To detect probe hybridisation, the slides were incubated for 30 min at room temperature with mouse-antibiotin monoclonal antibody, followed by biotinylated horse-anti-mouse, and finally with the avidin–biotin–peroxidase complex (Vector Laboratories, Burlingame, CA, USA). Finally, 0.1 M diaminobenzidine (Sigma) in PBS containing 0.03% H_2_O_2_ was applied for visualisation of the peroxides' activity. To improve identification of the individual nuclei, bright-field microscopy was combined with fluorescent nuclear counterstaining using 4,6-diamino-phenylindol (DAPI, Sigma). The samples were evaluated with a Leica DMBRE microscope (Leica Mikroskopie & Systeme GmbH, Wetzlar, Germany). The number of signals per nucleus was counted in at least 200 nonoverlapping nuclei. Trisomy/polysomy (gain) was defined as >5% of nuclei containing three or more signals, and monosomy (loss) as >25% of nuclei with none or one signal per nucleus. The tumours were classified as aneusomic when gain or loss of at least one of the chromosomes was detected. The other A II were classified disomic. As detected by ISH, 32 (68%) of the tumours showed aneusomy for one or more of the chromosomes investigated. The most frequent aberration was aneusomy for chromosome 7 in 31 (66%) of A II. Aneusomy for chromosome 1 was detected in 24 (51%) and aneusomy for chromosome 10 in 25 (53%) of the samples. Monosomy 10 was only detected in two samples (4%), which also showed trisomy/polysomy 7. The data of the individual chromosomes are described in detail elsewhere (Wessels *et al*, 2002).

### Statistical analyses

The following factors were considered as possible prognostic parameters for survival: patient age, sex, presenting symptoms, preoperative duration of symptoms, tumour location, neurosurgical procedure, timing of radiotherapy, proliferation index, and chromosomal status.

The. influence of these factors on survival was tested by univariate analysis using log-rank tests. Possible prognostic factors from the univariate analyses with *P*-values less than 0.10 (according to recommendation in the literature (Hosmer and Lemeshow, 2000)) were entered into the multivariate analyses using a forward stepwise method in order to identify independent prognostic parameters (Cox, 1972). Univariate and multivariate associations between factors and survival were assessed using a Cox regression model. Subsequently, tumour-related factors were used for analysis in age-stratified groups.

## RESULTS

**Figure 1:**
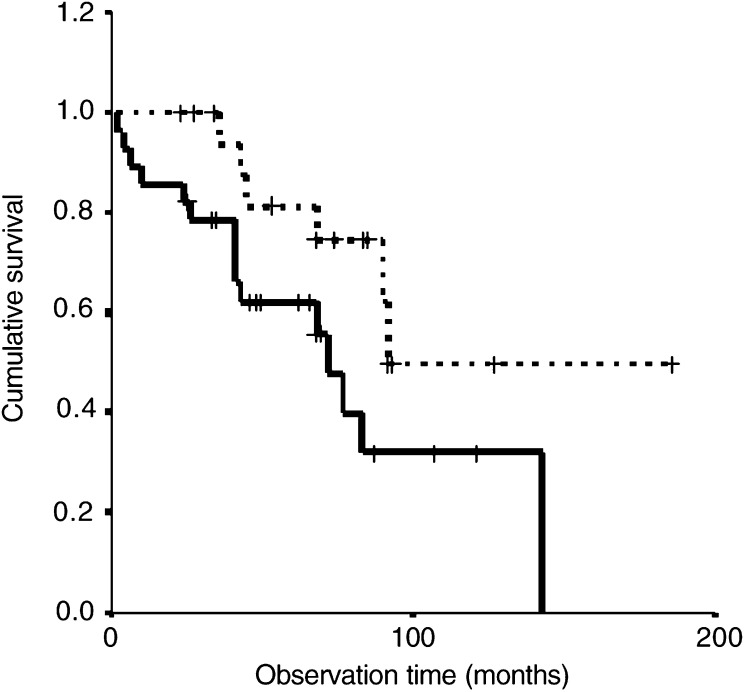
Association between patient age (18–34 *vs* ⩾35 years) and survival in A II. Kaplan–Meier, *P*-value = 0.05, log-rank. **··········** 18–34 years (*n*=19). **———** ⩾35 years (*n*=28).

The median survival interval for patients with astrocytoma grade II as estimated by the Kaplan–Meier method was 90 months (95% confidence interval 72–108 months). When determining the influence of age on the period of survival after the first diagnosis of A II, it becomes obvious that the group aged ⩾35 years at the time of diagnosis exhibits a significantly shorter survival period as compared to the 18–34 year-old patient group (Figure 1

; log-rank; *P*-value=0.05). Other factors significantly associated with shorter survival were focal neurological deficit at presentation, proliferation (Ki-67) index >1%, and aneusomy. Upon multivariate analysis proliferation index >1%, patient age ⩾35 years, and aneusomy were independently correlated with shorter survival (Table 1

**Table 1:** Unfavourable prognostic factors for patients with astrocytoma grade II

**Parameter**	**Hazard ratio (95% CI)^a^**	***P*-value**
*Univariate analysis*		
Proliferation index >1% (*n*=29)	3.63 (1.22–10.82)	0.02
Focal neurological deficit (*n*=13)	2.78 (1.12–6.90)	0.03
Aneusomy (*n*=32)	3.29 (0.97–11.20)	0.06
Patient age ⩾35 years (*n*=28)	2.52 (0.97–6.58)	0.06

*Multivariate analysis*		
Proliferation index >1% (n=29)	4.81 (1.50–15.38)	0.01
Patient age ⩾35 years (*n*=28)	3.01 (1.13–7.99)	0.03
Aneusomy (*n*=32)	3.66 (1.06–12.62)	0.04

a CI=confidence interval.

).

**Figure 2:**
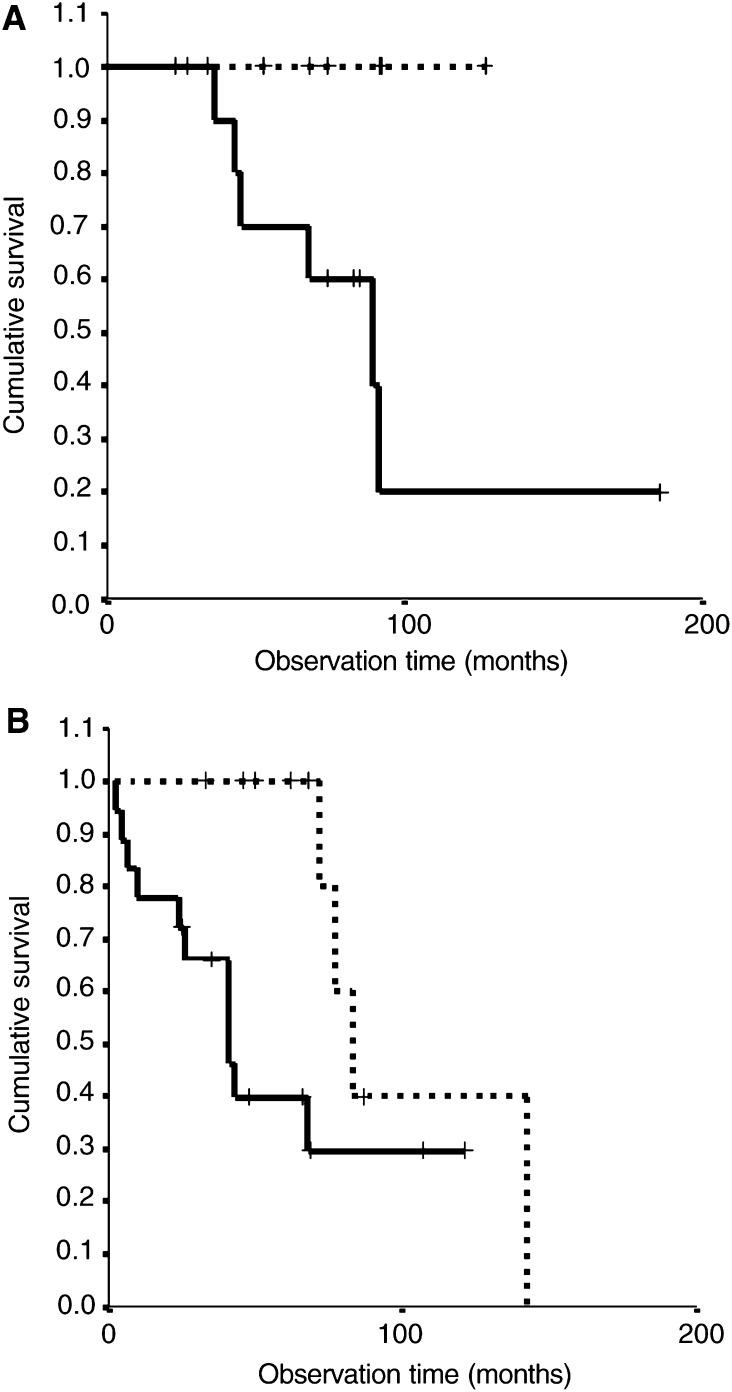
High proliferation (Ki-67) index in A II is associated with shorter survival in (**A**) patients aged 18–34 (*P*-value=0.02) and (**B**) patients ⩾35 years (*P*-value=0.03). Kaplan–Meier; pooled log-rank test, *P*-value=0.002. **··········** Proliferation index ⩽1% (*n*=18). **——** Proliferation index >1% (*n*=29).

To investigate the interactions between the proliferation index and aneusomy with age, we analysed the influence of these two factors on survival stratified for age. When correlating the proliferation index to survival in the two age groups separately, it appears that in the group of 18–34-year-old patients, a clearcut distinction can be made between those with a proliferation index ⩽1%, showing long-term survival, and those with a proliferation index >1%, showing more progressive AII (Figure 2A

; log-rank; *P*-value =0.02). Also in the ⩾35-year-old patients, the proliferation index proved to have additional prognostic value, although less apparent as compared to the younger group (Figure 2B; *P*-value=0.03).

**Figure 3:**
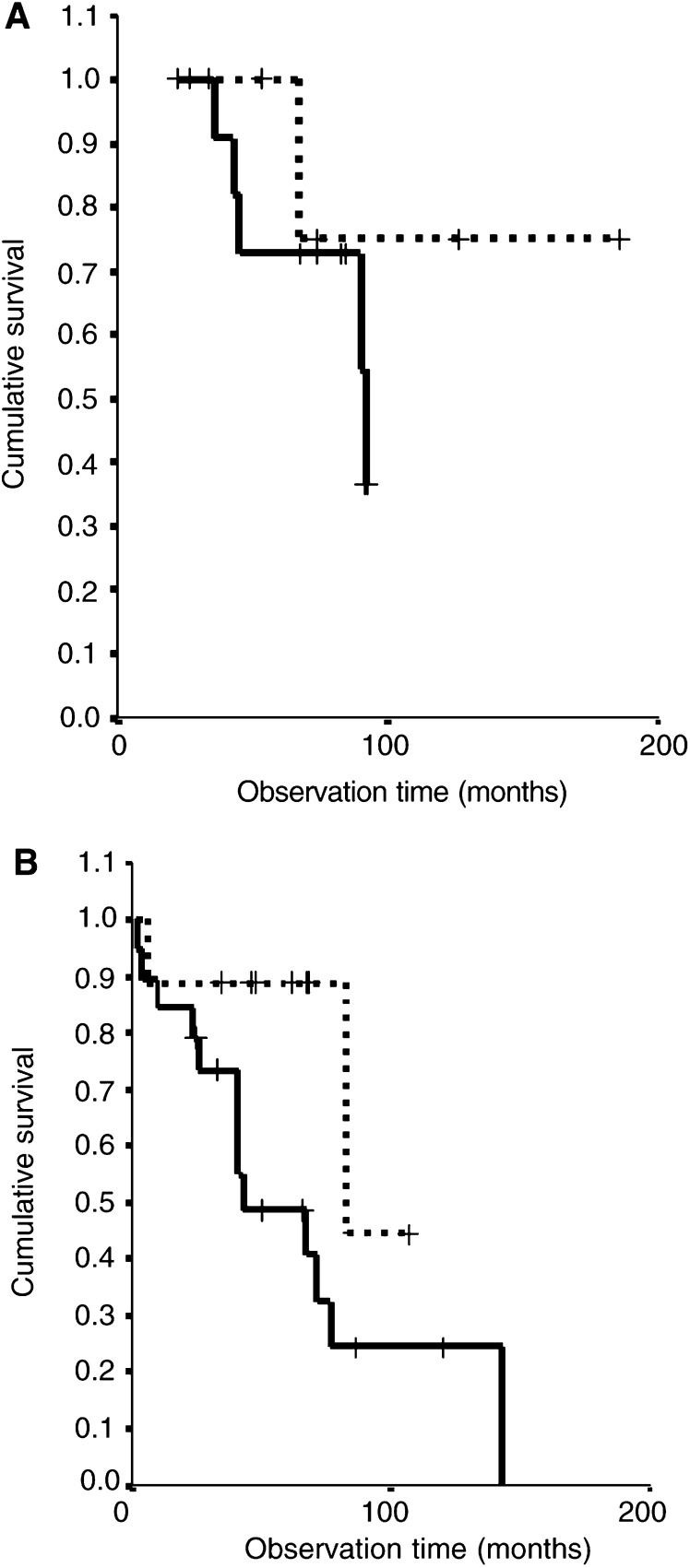
Relation of aneusomy with shorter survival in (**A**) A II patients aged 18–34 (*P*-value=0.36) and (**B**) patients ⩾35 years (*P*-value=0.09). Kaplan–Meier; pooled log-rank test, *P*=0.05. **··········** Disomy (*n*=15). **——** Aneusomy (*n*=32).

Using the ISH protocol with probes for chromosomes 1,7, and 10, cases of A II with an apparently normal (disomic) chromosomal content can be separated from aneusomic cases. In the whole group of patients with A II the detection of aneusomy has additional value in distinguishing between rapid and slow progressive A II. In the stratified age groups an aberrant chromosomal constitution is not associated with shorter survival in both the 18–34-year-old (Figure 3A

; *P*-value=0.36) and the ⩾35-year-old (Figure 3B; *P*-value=0.09) groups.

**Figure 4:**
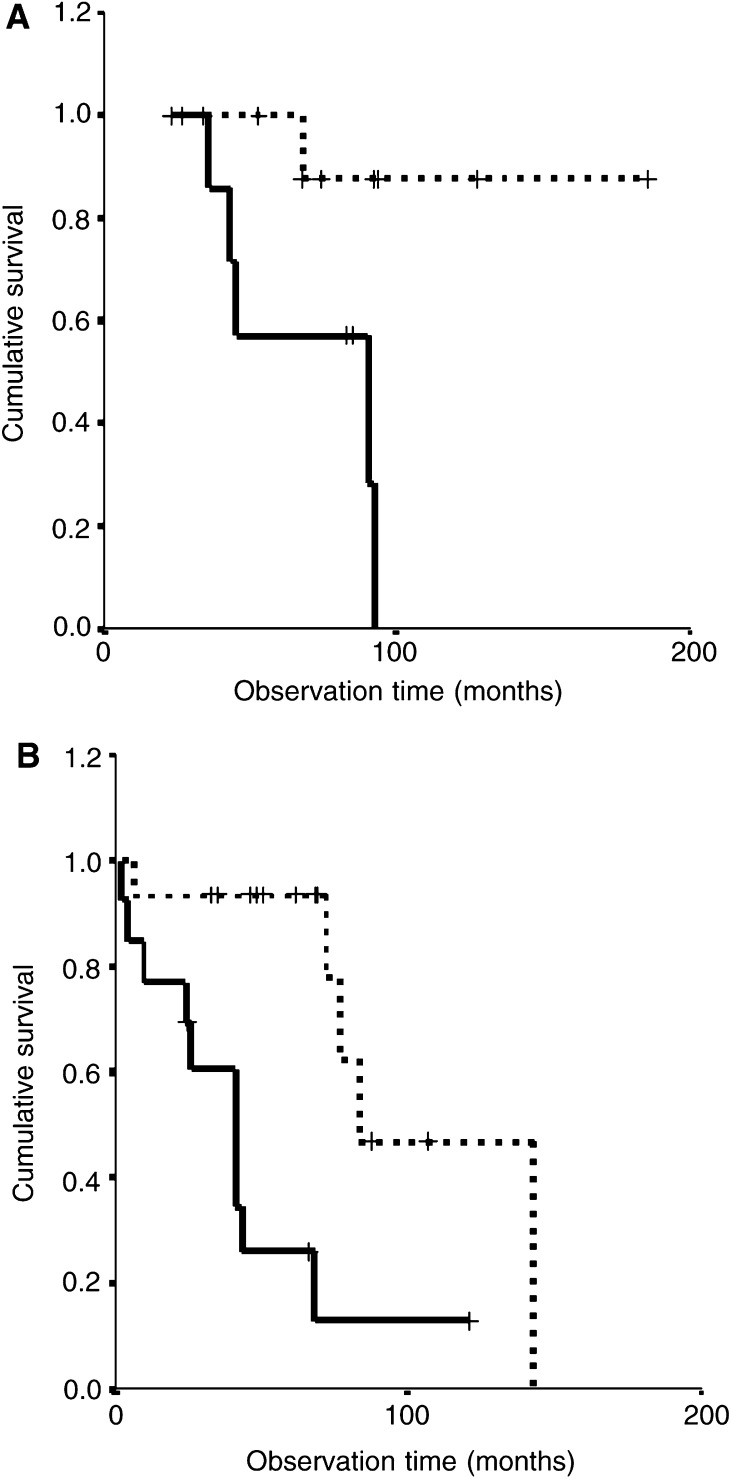
The combination of both high proliferation (Ki-67) index and aneusomy is strongly associated with a shorter survival in A II patients (**A**) aged 18–34 (*P*=0.01) and (**B**) aged ⩾35 years (*P*=0.001). Kaplan–Meier; pooled log-rank test, *P*<0.0001.**··········** Proliferation index ⩽1% or Disomy (*n*=26). **——** Proliferation index >1% and Aneusomy (*n*=21).

However, the combination of a high proliferation index and aneusomy very accurately identified patients with an unfavourable outcome in both the 18–34-year-old (Figure 4A

; *P*-value=0.01) and the ⩾35-year-old patient groups (Figure 4B; *P*-value=0.001). The combination of both factors reclassified three (27%) of 11 patients of 18–34 years into the group with relatively good prognosis (compare Figures 4A to 2A). In five of 18 patients in the ⩾35 years group, the high proliferation index with disomy now correlated with a more favourable course (compare Figures 4B to 2B).

Figure 5

**Figure 5:**
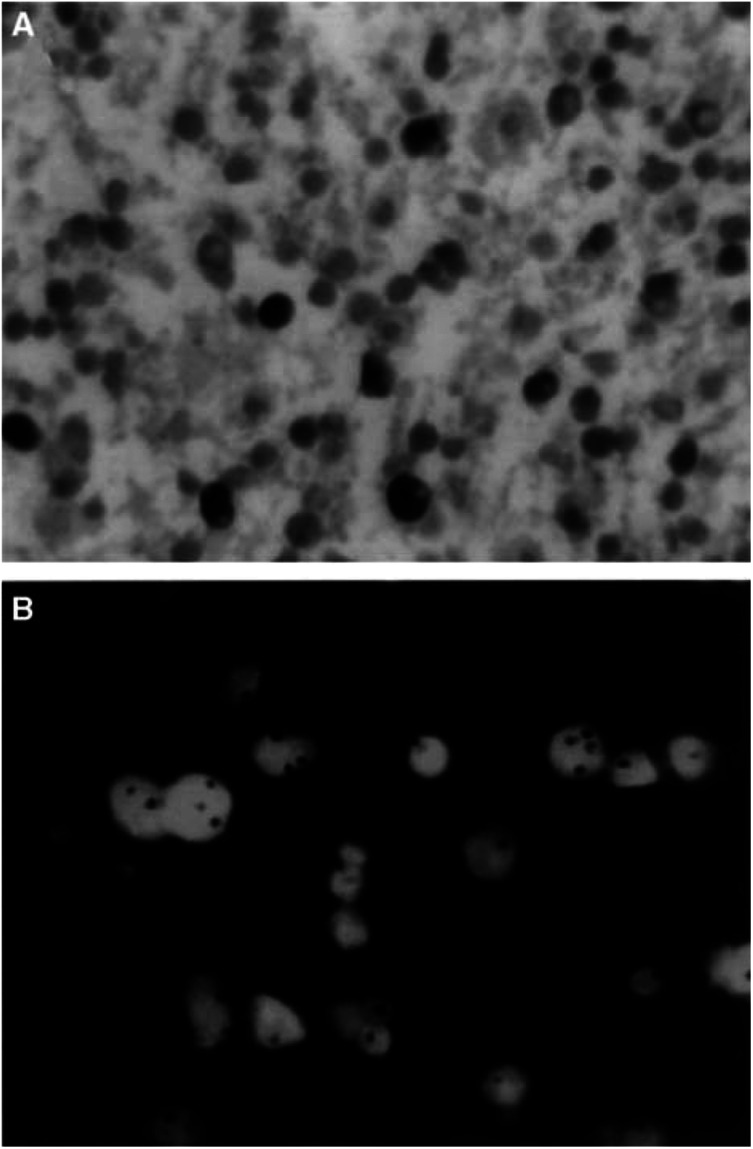
Astocytoma grade II in a patient aged 25 years with a high proliferation (Ki-67) index (**A**), and frequent nuclei with trisomy for chromosome 7, detected by ISH (**B**). Counter-staining with DAPI in (**B**). Magnifications × 800.

illustrates a case of A II, localised in the frontal lobe of a 25-year-old patient with epileptic seizures as the sole manifestation. The proliferation (Ki-67) index was 4.5% and a high percentage (35%) of nuclei with trisomy for chromosome 7 was detected by ISH. Despite the favourable clinical features (young age, no focal deficit), the patient rapidly progressed to astrocytoma grade IV, and the survival interval after histological diagnosis was only 36 months.

## DISCUSSION

A ‘wait and see’ policy has been propagated for patients under 35 years of age, suffering from epileptic seizures and radiological suspicion of astrocytoma grade II (A II) (Vecht, 1993). An important argument for not performing a stereotactic biopsy in these patients is that the histological diagnosis of A II does not alter treatment strategy. In the current study, we demonstrate that the detection of a high proliferative activity in combination with chromosomal aberrations identifies a subgroup of young A II patients with a rapid malignant course.

Our study confirms previous studies of A II showing the prognostic value of patient age and symptoms at presentation (Lote *et al*, 1997; Bauman *et al*, 1999). In multivariate analyses, only the age of patients at diagnosis remained significantly associated with survival as an independent clinical factor, when comparing patient group under and over 35 years of age. However, when including biological factors describing the proliferative capacity and genomic constitution of the lesion, subgroups of patients with a significantly shorter survival could be distinguished within these two separate age groups. In our study, immunostaining for the proliferation marker Ki-67 in over 1% of tumour cells showed the strongest association with survival of patients with A II. The proliferation (Ki-67) index has been described to increase with the grade of astrocytomas, although overlap between grades exists (Sallinen *et al*, 1994; Korkolopoulou *et al*, 1997). The additional value of the Ki-67 labelling protocol in A II was also demonstrated in two recent studies, although in these studies, no stratification for patient age was performed (McKeever *et al*, 1998; Heesters *et al*, 1999). We show a significant correlation of proliferation (Ki-67) index with survival for patients under 35 years, separating a group of young patients with a high proliferation index and a prognosis similar to that of patients >35 years. Immunolabelling of lesions from patients ⩾35 years of age identified a subgroup with a low proliferation index, correlating with a somewhat better prognosis. In the multivariate analysis, a proliferation (Ki-67) index >1% proved to be a strong independent prognosticator. This corroborates with a bromodeoxyuridine-(BrdU)- proliferation marker analysis of A II that also showed cell cycle activity in >1% of tumour cells to be associated with shorter survival (Ito *et al*, 1994). A possible drawback of the proliferation (Ki-67) index as prognosticator in A II is reflected by the wide variation of cutoff levels (between 2 and 10%) used in different studies (McKeever *et al*, 1998; Heesters *et al*, 1999; Fisher *et al*, 2002). In our series, the discriminative power of the proliferation (Ki-67) index diminished when using a higher cutoff level than 1% (data not shown). These differences in cutoff levels, among different studies, may be explained by differences in the immunohistochemical procedures applied, such as the method of antigen retrieval, the immunolabelling protocol, and by differences in the scoring criteria of (clustered) Ki-67-positive cells. Therefore, an additional, but independent prognosticator is needed.

The detection of numerical genomic aberrations has such additional prognostic value to the proliferation (Ki-67) index. For astrocytomas of all grades, it appears that certain genetic changes are associated with an unfavourable clinical course. Astrocytomas with chromosomal abnormalities as detected by karyotyping lead to a shorter survival as compared to astrocytomas without these abnormalities (Kimmel *et al*, 1992). An ISH study showed that monosomy for chromosome 10, harbouring the tumour suppressor gene PTEN/MMAC1, results in shorter survival (Cianciulli *et al*, 2000). However, in these series, the majority of tumours represented high-grade astrocytoma (grades III and IV). In A II, the most frequently reported genetic aberration is loss or mutation of the p53 gene, which, however, shows no association with clinical course (Kraus *et al*, 1994; al-Sarraj and Bridges, 1995).

One of the very few additional studies correlating genetic aberrations and clinical course includes a comparative genomic hybridisation (CGH) analysis, which showed that A II with rapid malignant progression exhibit a significantly higher number of chromosomal aberrations as compared to A II with an indolent behaviour (Sallinen *et al*, 1997). However, the sensitivity of this CGH technique is relatively low due to contamination with normal/reactive cells, which are often present in A II (Kallioniemi *et al*, 1994).

In contrast, the ISH procedure is routinely applicable to paraffin-embedded samples of central nervous system tumours (Arnoldus *et al*, 1992). Our study demonstrates that the detection of chromosomal aberrations by ISH, using a panel of probes for chromosomes 1, 7, and 10, offers an extra independent predictor for survival of patients with A II. After stratification for age, the presence of chromosomal aberrations alone is not significantly associated with shorter survival, which may be caused by the small size of both groups. However, in both age groups, the detection of aneusomy by ISH adds value to the proliferation index, in that increased proliferation in the presence of chromosomal aberrations is associated with a poor prognosis.

A correlation between aneusomy and proliferative activity was demonstrated in astrocytomas of all grades, containing aneusomies for chromosomes 7 and 10, particularly in Ki-67- positive cells (Steilen-Gimbel *et al*, 1996). This suggests that an accumulation of chromosomal aberrations in proliferating cells plays an important role in the early stages of astrocytoma progression. This also suggests that the combination of both these parameters is therefore very useful in identifying A II with a rapidly malignant clinical course.

Although imaging studies were used as adjunct to histology, A III/IV may also present as nonenhancing lesions (Chamberlain *et al*, 1988). Therefore, one could argue that in the stereotactic specimen, sampling error from high-grade astrocytomas may have biased the results. This is contradicted, however, by the relatively long median survival of 90 months, also compared to other A II studies (Leighton *et al*, 1997; McCormack *et al*, 1992). Another argument against underscoring of the tumours is given by the fact that only one of the biopsy specimens exhibited monosomy 10, which is characteristic at low-grade areas in high-grade astrocytomas (Cheng *et al*, 1999).

The additional value of genetic and biological parameters to the current histological WHO classification was also seen in the three gemistocytic variants of A II. Previous studies suggest that A II with high percentages (>60%) of gemistocytes, in fact, behave in a more similar manner to A III (Krouwer *et al*, 1991). Although two of our gemistocytic samples contained lower percentages of gemistocytes, the increased proliferation activity and the presence of aneusomy in all three samples was associated with a relatively short survival (range 41–43 months).

We postulate that identification of subtypes of astrocytomas on the basis of genetic and biological factors has additional prognostic value to the current histological classification. Tissue should be obtained in all patients with A II in order to assess the proliferation activity and, if possible, the presence of trisomy for chromosome 7. In the future, these markers may help in optimising treatment strategy, in particular in young patients with astrocytoma grade II, for whom optimal treatment is now controversial.
